# The association of *HLA/KIR* genes with non-small cell lung cancer (adenocarcinoma) in a Han Chinese population

**DOI:** 10.7150/jca.33566

**Published:** 2019-08-20

**Authors:** Yingfu Li, Shuyuan Liu, Chao Hong, Qianli Ma, Fang Tan, Chengxiu Liu, Piotr Kuśnierczyk, Chuanyin Li, Li Shi, Yufeng Yao

**Affiliations:** 1 Institute of Medical Biology, Chinese Academy of Medical Sciences & Peking Union Medical College, Kunming 650118, China; 2 Department of Geriatrics, The No.1 Affiliated Hospital of Kunming Medical University, Kunming 650032, China; 3 Department of Thoracic Surgery, The No.3 Affiliated Hospital of Kunming Medical University, Kunming 650118, China; 4 Laboratory of Immunogenetics and Tissue Immunology, Ludwik Hirszfeld Institute of Immunology and Experimental Therapy, Polish Academy of Sciences, Wrocław, Poland

**Keywords:** non-small cell lung cancer, *HLA*, * KIR*, *HLA/KIR* combination, Chinese Han population

## Abstract

The host immune system plays a crucial role in the surveillance, recognition and elimination of tumor cells. Recent studies found that Human lymphocyte antigen class I (*HLA I*) genes, Killer cell immunoglobulin-like receptor (*KIR*) genes and *HLA/KIR* combinations play a role in the defense against tumor cells. To evaluated the associations between *HLA I* genes, *KIR* genes and *HLA/KIR* combinations and non-small cell lung cancer (NSCLC) in a Chinese Han population, a total of 229 patients with NSCLC (adenocarcinoma) and 217 healthy individuals were studied. Our results showed that the HLA-C*08:01 allele occurred at a significantly higher frequency in the NSCLCs compared with the controls (*P*=0.034). The *HLA* haplotype frequencies bearing *HLA-A*, *-B*, and *-C* loci between the NSCLC and control groups were not different (*P*>0.05). And there were no differences in the *KIR* gene, genotype and haplotype frequencies between the NSCLC and control groups (*P*>0.05). Also, there were no differences between the *HLA/KIR* combinations in the *KIR3D* genes and *HLA-A3/A11*, *HLA-Bw4* ligands and *KIR2D* genes and HLA-C1/C2 ligands between the NSCLC and control groups (*P*>0.05). Our results indicate that the HLA-C*08:01 allele could be a risk factor for NSCLC (adenocarcinoma) in the Chinese Han population (OR=2.395; 95% CI: 1.359-4.221).

## Introduction

Lung cancer causes a large number of cancer-related deaths around the world, and its 5-year survival rate is approximately 15% 1. Among lung cancer cases, non-small cell lung cancer (NSCLC) accounts for approximately 80% of lung cancer cases 2. To date, more and more studies have proven that host genes play a key role in the susceptibility or development of NSCLC, especially host immune system genes.

The host immune system can recognize and eliminate tumor cells 3; this is mediated by CD8^+^ cytotoxic T cell adaptive immune responses. Human lymphocyte antigen class I molecules (HLA-I) play a key role in presenting tumor antigens for CD8^+^ cytotoxic T cells recognition 4. HLA-I molecules are encoded by *HLA I* genes; three classical transplantation *HLA* genes (*HLA-A, HLA-B* and *HLA-C*) have been observed to have multiple roles in immune regulation 5.

Natural killer (NK) cells are crucial components of the innate immune system, which is involved in the immune response to different diseases including cancers 6. Killer cell immunoglobulin-like receptors (KIRs) are present on NK cells, and located within the leukocyte receptor complex 7. To date, 16 *KIR* gene loci have been identified, including two pseudogenes (*KIR2DP1* and *KIR3DP1*), seven inhibitory *KIR* genes (*KIR2DL1, KIR2DL2, KIR2DL3, KIR 2DL5, KIR3DL1, KIR3DL2* and *KIR3DL3*), six activating genes (*KIR2D S1, KIR2DS2, KIR2DS3, KIR2DS4, KIR2DS5*, and *KIR3DS1*), and *KIR2DL4*, whose product may have both inhibitory and activating capacities 8-10.

HLA-I molecules and KIR serve as ligands and receptors, respectively, and their interplay is important for transmitting activating or inhibitory signals to regulate the function of NK cells 11. Their interactions are also a component of the innate immune system, which plays a role in the defense against tumor cells. Thus, the function of *HLA I* genes, *KIR* genes and their combinations are valuable for investigating associations with susceptibility or worse clinical outcomes in different types of cancer, such as kidney cancer, breast cancer, colorectal cancer and lung cancer 12-18.

To investigate the role of genetic variations in *HLA I* genes, *KIR* genes and *HLA/KIR* combinations in the susceptibility or development of NSCLC, we evaluated the association of *HLA I* genes, *KIR* genes and *HLA/KIR* combinations with NSCLC (adenocarcinoma) in a Chinese Han population in this study.

## Material and Methods

### Ethical approval and informed consent

All procedures were in accordance with the ethical standards of the responsible committee on human experimentation and with the Helsinki Declaration of 1964, which was revised in 2013. All experimental protocols used in this study were approved by the Institutional Review Boards of the No. 3 Affiliated Hospitals of Kunming Medical University. All participants provided written informed consent.

### Subjects

From December 2013 to January 2016, we selected 229 patients (134 males and 95 females) as the case group who were diagnosed with NSCLC and adenocarcinoma based on pathomorphological reports at the No. 3 Affiliated Hospitals of Kunming Medical University. The histological type and pathologic stage of the lung cancer was identified according to the World Health Organization (WHO 2004) classifications and the International System for Staging Lung Cancer 19. The NSCLC patient inclusion criteria: 1) the patients were aged ≥18 years with histologically and pathologically diagnosed NSCLC; 2) the patients were diagnosed with adenocarcinoma; 3) the patients had not received preoperative neoadjuvant therapies (including chemotherapy and radiotherapy). The criteria for the exclusion was 1) the patients with a prior history of primary cancer other than lung cancer; 2) the patients with small cell lung cancer; 3) the patient with malignant tumors except lung cancer; 4) the patients receiving radiotherapy or chemotherapy, and unclear pathological diagnosis. Clinical characteristics and data, such as sex, age, family history of cancer, and histological type of cancer, were obtained. The healthy controls included 217 subjects (143 males and 74 females) were recruited from a population undergoing routine examinations at the No.3 Affiliated Hospitals of Kunming Medical University. The controls underwent clinical examinations and did not have any cancer, or family history of NSCLC, or respiratory diseases and matched to the cases by age and gender. All participants (NSCLC patients and healthy controls) were unrelated Chinese Han individuals.

### HLA I genes (HLA-A, -B and -C) genotyping

Blood samples were collected and genomic DNA was extracted from peripheral lymphocytes using the QIAamp Blood Kit (Qiagen, Hilden, Germany) according to the manufacturer's protocol. *HLA-A, -B*, and *-C* genes were genotyped with the NGS sequencing-based *HLA* typing using the NGSgoR Illumina Miseq workflow (Illumina, Inc. San Diego, CA, USA) according to the manufacturer's instructions. Briefly, the NGSgo-AmpX kit was first used to perform *HLA* locus-specific (*HLA-A*, *-B*, and *-C*) amplification. Then, the large amplicons were randomly fragmented to 300-800 bp prior to adapter ligation to ensure that the whole gene was evenly covered by numerous overlapping reads. The end-repair and dA-tailing were completed in the same step. After fragmentation, adapter ligation was performed, followed by a second clonal amplification and then sequencing. During sequencing, millions of reads were generated from multiple samples and multiple loci simultaneously. The NGS engine software package (Illumina) was used to determine the HLA genotypes for the individual samples and loci in an easy and user-friendly manner based on NGS data using the following three steps: (1) Alignment, (2) Haplotype phasing, and (3) Genotype determination.

### KIR gene genotyping

In the current study, sixteen *KIR* genes (excluding 3DP1) 20 were genotyped using multiplex PCR sequence-specific priming (PCR-SSP) and identified by electrophoresis via agarose gels as described previously 21. Two sets of primers were designed for each of the sixteen KIR genes and the presence of a gene was detected only when both were amplified.

### Statistical analysis

The *HLA-A, -B* and *-C* allele frequencies were calculated with the Pypop and PyHLA software based on the genotyping results 22-24. The χ² test was used to determine differences in the allele frequencies between the NSCLC and healthy control groups. The odds ratios (OR) and associated 95% confidence intervals (CIs) were also calculated for allele-specific risks. The Hardy-Weinberg equilibrium was assessed using the Guo and Thompson method 25. The *HLA-A, -B* and *-C* haplotypes were constructed and calculated using the expectation-maximization algorithm based on the genotyping results.

For *KIR* loci, the frequency of each *KIR* gene was determined by direct counting. The genotypes and haplotypes were defined by referring to the Allele Frequencies website (http://www.allelefrequencies.net). The χ² test was used to determine differences in gene, genotype and haplotype frequencies between the NSCLC and healthy control groups.

For *HLA/KIR* combinations, the inhibitory *KIR3DL1* recognizes *HLA-B Bw4* and *KIR3DL2* binds to *HLA-A3* and *HLA-A11* 26-28. Based on the dimorphism in the epitope at position 80, all HLA-C alleles can be divided into the following two groups: (1) the C1 group carrying asparagine and (2) the C2 group carrying lysine. The C1 group consists of *HLA-Cw1*,* -Cw3*,* -Cw7*, *-Cw8*, *-Cw12*, -*Cw13*, and *-Cw14*. The C2 group consists of *HLA-Cw2*, *-Cw4*, *-Cw5*, *-Cw6*,* -Cw15*, and *-Cw17*. The HLA-C1 ligands bind to KIR2DL2, KIR2DL3 and KIR2DS2 receptors, whereas HLA-C2 ligands bind to KIR2DL1 and KIR2DS1 26, 29, 30. Thus, the HLA-A3/A11, Bw4, C1 and C2 groups were also used to analyze the HLA/KIR combinations. The differences in the HLA/KIR combination frequencies between the NSCLC and healthy control groups were determined by the χ² test.

The false discovery rate (FDR) correction was used for the multiple comparisons 22, 31, and adjusted *P* value less than 0.05 was considered statistically significant.

## Results

### Subject characteristics

Table 1 lists the characteristics of the enrolled subjects. There were no age or gender differences between the NSCLC and healthy control groups (*P*>0.05).

### Association of HLA I genes and their haplotypes with NSCLC

The allele frequencies for *HLA-A*, *-B* and *-C* in the NSCLC and healthy control groups are listed in Table 2. The genotype frequencies for *HLA-A*, *-B* and *-C* were in Hardy-Weinberg equilibrium for both the NSCLC and healthy control groups (*P*>0.05). The frequencies of the HLA-A*02:06, B*13:01, B*40:01, and C*08:01 alleles were different between the NSCLC and healthy control groups (*P*<0.05). However, after FDR correction, only HLA-C*08:01 occurred at a significantly higher frequency in the NSCLC group compared with the healthy control group (*P*=0.034, OR=2.395; 95% CI: 1.359-4.221). Additionally, there is no differentiation of the susceptibility between male and female (*P*=0.482). The *HLA* haplotype analysis (>1%) bearing HLA-A, -B, -C loci showed that the ten haplotypes (A*02:01-B*46:01-C*01:02, A*02:03-B*15:02-C*08:01, A*11:01-B*15:02-C*08:01, A*11:01-B*40:01-C*03:04, A*11:01-B*52:01-C*12:02, A*11:01-B*54:01-C*01:02, A*24:02-B*15:25-C*04:03, A*24:02-B*40:01-C*07:02, A*24:02-B*51:01-C*14:02 and A*33:03-B*44:03-C*07:01) were different between the NSCLC and healthy control groups (Table 3), however, the significance of differences faded after FDR correction (Table 3). In the subtype analysis, there were differences in frequencies of HLA-B*15:11 and haplotype A*02:01-B*15:11-C*03:03, A*11:01-B*38:02-C*07:02, A*2402-B*07:02-C*07:02 and A*24:02-B*46:01-C*01:02 between pathologic stages I+II and III+IV (data not shown). However, after FDR correction, there were no differences in the *HLA* alleles and haplotypes between pathologic stages I+II and III+IV (data not shown).

### Association of 16 KIR genes and their haplotypes with NSCLC

All 16 *KIR* genes were detected in the NSCLC and healthy control groups. The four framework loci, *KIR3DL3*, *KIR3DP1*, *KIR2DL4*, and *KIR3DL2*, were observed in every individual in the NSCLC and healthy control groups. The KIR gene (*KIR2DL2*, *KIR2DL5*, *KIR2DS2* and *KIR2DS3*) and genotype (5, 15, 23 and 64) frequencies were different in the NSCLC and healthy control groups (*P*<0.05). However, there were no significant differences in the *KIR* genes and genotypes between the NSCLC and healthy control groups after FDR correction (Tables 4 and 5). KIR haplotypes A and B showed no significant differences in the NSCLC and healthy control groups with frequencies of 0.459 vs. 0.525 for haplotype A and 0.541 vs. 0.475 for haplotype B, respectively (*P*>0.05) (Supplementary Table 1). In the subgroups analysis, only KIR genotype 9 showed differences between pathologic stages I+II and III+IV (data not shown). However, after FDR correction, there were no differences in the *KIR* gene, genotype and haplotype frequencies between pathologic stages I+II and III+IV (data not shown).

### Associations of HLA/KIR combinations with NSCLC

The distribution of the two *KIR3D* genes and their HLA-A3/A11 or HLA-B Bw4 ligands is given in Supplementary Table 2. There were no significant differences (*P*>0.05) in their combination frequencies between the NSCLC and healthy control groups. The distribution of the *KIR2D* genes and their HLA-C1/C2 ligands is given in Supplementary Table 2. The results for the *KIR2D* genes and their HLA-C1/C2 ligands showed that there were no significant differences in the frequencies between the NSCLC and healthy control groups (*P*>0.05).

Recent evidence has demonstrated associations between *HLA I* genes, *KIR* genes and *HLA/KIR* combinations and autoimmune disease, infectious disease and cancers 12-18, 32-37. In this study, we examined the role of genetic variations in *HLA I* genes, *KIR* genes and *HLA/KIR* combinations in the development of NSCLC in a Chinese Han population.

NSCLC is one of the most immunogenic tumors, and its tumor antigens can potentially be recognized by CTLs and CD8^+^ cytotoxic T cells, which mediate antitumor responses 38. In 2010, Yang *et al* 39 investigated HLA-A*02:01, A*26:01, B*15:18, and B*38:02 were associated with lung cancer in a Chinese Han population from North China. In the current study, we found that the frequencies of HLA-A*02:06, B*13:01 and B*40:01 were lower in the NSCLC group than in the healthy control group (0.018 vs. 0.053, 0.046 vs. 0.090 and 0.081 vs. 0.124, respectively) (Table 2), though there were no differences between the two groups after FDR correction. Nevertheless, our results showed that HLA-C*08:01 occurred at a significantly higher frequency in the NSCLC group compared with the healthy control group and showed a significant difference between the two groups after FDR correction (OR=2.395; 95% CI: 1.359-4.221). One of the reasons for this discrepancy in the results between the Yang study and our study could be related to differences in sample sizes and pathomorphological type. In the current study, we enrolled 229 NSCLC patients and 217 healthy individuals, while only 100 lung cancer patients were included in Yang's study (81 NSCLC and 19 small cell lung cancer patients). Additionally, our NSCLC group only included adenocarcinoma patients, while Yang's study included lung cancer patients who were not further classified by pathomorphological type. Another reason might be the genetic differences between the North Han and Yunnan Han population. In 2015, Zhou *et al*. investigated the distribution of HLA allele and haplotype frequencies in the Chinese population, and found there were regional differences in the HLA diversities in China 40. Moreover, our previous study also showed Han Chinese populations were divided into northern and southern clusters based on *HLA-A*, *-B*, and *-DRB1* allele frequencies using phylogenetic tree and multidimensional scaling analyses 41. The Han population from Yunnan is situated between the northern and southern clusters and showed different genetic characteristics from the Northern Han population 41. The third reason could be because there was no correction for multiple comparisons in Yang's study, but we used FDR for multiple comparisons in the current study. In 2009, Nagata *et al* 42 reported that HLA-A*02 and HLA-A*24 could be prognostic factors for Japanese patients with NSCLC. Then, Bulut *et al* 43 investigated that HLA-A*02 was an independent risk factor for lymph node and distant metastases in patients with NSCLC in Turkish populations. Additionally, they also found that HLA-A*26 appeared to be a protective allele against metastases. However, we did not observe these differences in the *HLA I* gene allele and haplotype frequencies between pathologic stages I+II and III+IV after multiple comparison correction.

To escape CD8^+^ cytotoxic T cell recognition, tumor cells must eliminate HLA I molecules by downregulating their expression at the cell surface 44. However, if HLA expression is completely lacking, NK cells will attack and kill tumor cells. Thus, variations in *KIR* genes have been thought as a major risk for developing cancer 45, 46. In 2010, Al Omar *et al* 17 reported that there were no significant differences in the proportion of patients with different *KIR* genes, genotypes and haplotypes among patients with solid tumors (NSCLC, small-cell lung cancer, colon cancer and kidney cancer). Then, Yu *et al* reported the *KIR* gene frequency showed no differences between NSCLC and healthy controls in a Chinese Han population 14. Also Wiśniewski *et al* 13 did not find such differences in Polish Caucasians. Our results were similar to the Al Omar, Yu and Wiśniewski results in that we also found no differences in the *KIR* gene, genotype and haplotype frequencies. We also found that there were no differences in the *KIR* gene, genotype and haplotype frequencies between pathologic stages I+II and III+IV after multiple comparison correction.

## Discussion

Several studies have reported that certain *HLA/KIR* combinations are associated with susceptibility and worse clinical outcomes and response to treatment in several types of cancer, including breast cancer, colorectal cancer and lung cancer 12-18. In 2010, Al Omar *et al* performed an association study on *HLA/KIR* combinations with NSCLC; they found that NSCLC patients showed a significant increase in the frequency of *KIR2DL1-C2* and decrease in the frequency of *KIR2DL3-C1* in homozygotes 17. However, in the current study, our results did not show any differences in the *HLA/KIR* frequencies between the NSCLC and healthy controls in the Chinese Han population, which was similar to a report by Wiśniewski for Polish Caucasians 13. These authors observed only that *HLA-C* heterozygotic genotypes encoding both *C1* and *C2* ligand epitopes for *KIR2DL2/3* and *KIR2DL1* inhibitory receptors, respectively, were less frequent in patients than in controls, whereas opposite was true for *C1C1* and *C2C2* homozygotes. However, this association of KIR ligands was independent from their respective receptors or any other *KIR* genes 13. In the present report, even when subgroup analysis was performed, there were no differences in the *HLA/KIR* combinations between pathologic stages I+II and III+IV.

Nevertheless, Wiśniewski *et al* found that NSCLC patients positive for the *KIR2DL2* and *KIR2DS2* genes and homozygous for *C1* were 6 times more likely to respond to treatment than those with other genotypes (*P*=0.034). In accordance with these results, patients with the *KIR2DL2^+^/KIR2DS2^+^*-*C1C1* genotype survived longer than others (*P*=0.009) 13. However, Yu *et al* did not observe any relationship between *HLA/KIR* combinations and response to treatment in NSCLC in a Chinese Han population 14. Here, we did not examine the response to NSCLC treatment, which is one of limitations of the current study; thus, we could not compare our results with others. However, we did not observe any differences in the *HLA/KIR* combinations between pathologic stages I+II and III+IV.

We used the association studies to investigate the relationship between *HLA/KIR* and NSCLC in the current study. One of the disadvantages in the association study is the high probability of false positive. Several factors influence the rate of false positive, such as population heterogeneity and stratification, and multiple testing. In order to avoid population heterogeneity and stratification, in this current study, we recruited the Han Chinese from the same region only, in addition, we restricted the pathomorphological to adenocarcinoma and made a series of inclusion and exclusion criteria for the case and control group which matched each other in gender and age. For the multiple testing, we used FDR correction for multiple comparisons and considered adjusted P value less than 0.05 statistically. FDR is a method of conceptualizing the rate of type I error in null hypothesis testing when conducting multiple comparisons. FDR-controlling procedures provide less stringent control of Type I errors compared to family-wise error rate (FWER) controlling procedures and have greater power at the cost of increased numbers of Type I errors 31.

Another limitation of the current study was smoking history, which is a key risk factor for NSCLC and was not included in the data derived from the healthy control individuals in the current study. Although about 85-90% of lung cancer cases are smokers, the frequency of this malignancy among non-smokers seems to rise 47. Several genetic polymorphisms have been recently found to be associated with lung cancer among non-smoking Chinese women 48, 49, including those not exposed to cooking oil fume 50, 51. For Brasilian lung cancer patients (mostly with adenocarcinomas), different rates of EFGR and KRAS mutations were found in smokers and non-smokers 52. The lack of smoking status for our healthy control individuals and patients makes it difficult to perform analyses that include such exposure variables and to perform a gene-smoking interaction analysis. Therefore, we are planning to recruit sufficient numbers of non-smoker NSCLC patients and comparable number of non-smoker controls to examine genetic risk factors independent of smoking.

Our interesting finding that HLA-C*08:01 allele is associated with NSCLC in Yunnan Han Chinese is novel, as such a detailed study has not been so far performed in this or other populations (Pubmed search, 3 January, 2019). In addition, this association could hardly be detected, for example, in Caucasians, as HLA-C*08:01 is very rare there, in contrast to HLA-C*08:02, more frequent in Europeans but extremely rare in Chinese (www.allelefrequencies.net). In 2016, Eric Tran *et al* reported that they identified a polyclonal CD8^+^ T-cell response against mutant KRAS G12D in tumor-infiltrating lymphocytes, and observed regression of lung metastases after the infusion of HLA-C*08:02-restricted tumor-infiltrating lymphocytes that were composed of four different T-cell clonotypes that specifically targeted KRAS G12D 53. The molecules encoded by these two alleles differ to some extent in their peptide-binding motifs and therefore might also differ in their roles in the immune surveillance of cancer 54.

## Conclusion

In the current study, we performed an association study between *HLA I* genes, *KIR* genes and *HLA/KIR* combinations and NSCLC in a Chinese Han population and found that HLA-C*08:01 is a risk factor for NSCLC (adenocarcinoma). In the future, larger scale studies are needed to better clarify and examine the association between *HLA I* genes, *KIR* genes and *HLA/KIR* combinations and NSCLC susceptibility, resistance and disease progression.

## Supplementary Material

Supplementary tables.

## Figures and Tables

**Table 1:** Clinical characteristics of the subjects enrolled in the present study

	NSCLC (n=229)	Control (n=217)	*P* value
**N**	229	217	446
**Ages ( years )**	54.85±11.20	54.42±7.46	0.634
**Sex ( M/F )**	134/95	143/74	0.108
**Adenocarcinoma (AD)**	229		
**Clinical stage**			
I	38		
II	39		
III	66		
IV	86		

**Notes:** Data are mean±SD

**Table 2:** *HLA-A*, *HLA-B*, and *HLA-C* allele frequencies in NSCLC group (n=229) and healthy control group (n=217)

Allele	NSCLC (freq)	Control (freq)	*P* value	OR
A*01:01	0.018	0.023	0.851	0.754
A*02:01	0.118	0.085	0.721	1.434
A*02:03	0.066	0.042	0.721	1.620
A*02:06	0.018	0.053	0.111	0.318
A*02:07	0.107	0.097	0.851	1.118
A*02:10	0.002	0.002	1.000	0.948
A*03:01	0.011	0.028	0.721	0.388
A*03:02	0.002	0.002	1.000	0.948
A*11:01	0.273	0.277	1.000	0.982
A*11:02	0.004	0.007	0.851	0.630
A*24:02	0.168	0.194	0.851	0.842
A*24:03	0.009	0.005	0.851	1.903
A*24:07	0.013	0.007	0.851	1.907
A*26:01	0.031	0.018	0.851	1.679
A*29:01	0.009	0.002	0.851	3.815
A*30:01	0.031	0.025	0.851	1.213
A*31:01	0.031	0.018	0.851	1.679
A*32:01	0.007	0.012	0.851	0.566
A*33:03	0.076	0.088	0.851	0.862
A*68:01	0.002	0.007	0.851	0.314
A*69:01	0.004	0.005	1.000	0.947
B*07:02	0.015	0.012	1.000	1.332
B*07:05	0.011	0.002	0.876	4.779
B*08:01	0.004	0.005	1.000	0.947
B*13:01	0.046	0.090	0.373	0.487
B*13:02	0.039	0.023	0.876	1.735
B*15:01	0.048	0.042	1.000	1.166
B*15:02	0.070	0.042	0.726	1.736
B*15:07	0.002	0.002	1.000	0.948
B*15:11	0.013	0.018	1.000	0.707
B*15:12	0.015	0.007	0.890	2.230
B*15:18	0.004	0.002	1.000	1.899
B*15:25	0.018	0.018	1.000	0.947
B*15:27	0.004	0.007	1.000	0.630
B*27:04	0.011	0.014	1.000	0.787
B*35:01	0.033	0.042	1.000	0.783
B*35:05	0.013	0.009	1.000	1.427
B*37:01	0.007	0.009	1.000	0.709
B*38:02	0.042	0.030	0.890	1.402
B*39:01	0.028	0.018	0.890	1.556
B*40:01	0.081	0.124	0.619	0.619
B*40:02	0.022	0.012	0.876	1.915
B*40:06	0.028	0.016	0.876	1.782
B*44:02	0.004	0.014	0.876	0.313
B*44:03	0.015	0.028	0.876	0.546
B*46:01	0.157	0.152	1.000	1.040
B*48:01	0.007	0.021	0.726	0.311
B*50:01	0.004	0.002	1.000	1.899
B*51:01	0.055	0.032	0.876	1.732
B*51:02	0.011	0.007	1.000	1.586
B*52:01	0.033	0.035	1.000	0.946
B*54:01	0.046	0.044	1.000	1.050
B*55:02	0.024	0.028	1.000	0.865
B*56:01	0.004	0.002	1.000	1.899
B*57:01	0.004	0.007	1.000	0.630
B*58:01	0.042	0.058	0.876	0.708
C*01:02	0.240	0.217	0.851	1.143
C*03:02	0.044	0.062	0.851	0.688
C*03:03	0.063	0.065	1.000	0.980
C*03:04	0.085	0.124	0.499	0.655
C*04:01	0.055	0.060	1.000	0.906
C*04:03	0.020	0.032	0.851	0.601
C*05:01	0.004	0.005	1.000	0.947
C*06:02	0.055	0.046	1.000	1.195
C*07:02	0.173	0.148	0.851	1.205
C*07:04	0.009	0.002	0.851	3.815
C*08:01	0.094	0.042	0.034	2.395
C*12:02	0.026	0.039	0.851	0.660
C*12:03	0.022	0.023	1.000	0.946
C*14:02	0.039	0.037	1.000	1.069
C*14:03	0.004	0.002	1.000	1.899
C*15:02	0.026	0.023	1.000	1.141

**Notes:** The *P* value is corrected by FDR correction

**Table 3:** Common *HLA A-B-C* haplotypes frequencies in NSCLC group (n=229) and healthy control group (n=217)

Haplotype	NSCLC (freq)	Control (freq)	*P* value
A*02:01-B*15:11-C*03:03	0.011	0.007	>0.05
A*02:01-B*40:01-C*03:04	0.008	0.010	>0.05
A*02:01-B*46:01-C*01:02	0.000	0.012	>0.05
A*02:03-B*15:02-C*08:01	0.015	0.000	>0.05
A*02:03-B*38:02-C*07:02	0.014	0.009	>0.05
A*02:07-B*46:01-C*01:02	0.090	0.075	>0.05
A*11:01-B*13:01-C*03:04	0.027	0.035	>0.05
A*11:01-B*15:01-C*04:01	0.012	0.009	>0.05
A*11:01-B*15:02-C*08:01	0.043	0.014	>0.05
A*11:01-B*38:02-C*07:02	0.013	0.007	>0.05
A*11:01-B*40:01-C*03:04	0.004	0.019	>0.05
A*11:01-B*40:01-C*07:02	0.033	0.023	>0.05
A*11:01-B*46:01-C*01:02	0.024	0.032	>0.05
A*11:01-B*51:01-C*14:02	0.018	0.014	>0.05
A*11:01-B*52:01-C*12:02	0.000	0.012	>0.05
A*11:01-B*54:01-C*01:02	0.000	0.013	>0.05
A*11:01-B*55:02-C*01:02	0.013	0.008	>0.05
A*24:02-B*13:01-C*03:04	0.010	0.015	>0.05
A*24:02-B*15:25-C*04:03	0.003	0.016	>0.05
A*24:02-B*38:02-C*07:02	0.013	0.002	>0.05
A*24:02-B*39:01-C*07:02	0.010	0.005	>0.05
A*24:02-B*40:01-C*03:04	0.012	0.002	>0.05
A*24:02-B*40:01-C*07:02	0.000	0.018	>0.05
A*24:02-B*46:01-C*01:02	0.030	0.015	>0.05
A*24:02-B*51:01-C*14:02	0.010	0.000	>0.05
A*24:02-B*54:01-C*01:02	0.018	0.017	>0.05
A*30:01-B*13:02-C*06:02	0.024	0.021	>0.05
A*33:03-B*44:03-C*07:01	0.000	0.012	>0.05
A*33:03-B*58:01-C*03:02	0.037	0.044	>0.05

**Notes:** The *P* value is corrected by FDR correction

**Table 4:** *KIR* genes frequencies in NSCLC group (n=229) and healthy control group (n=217)

KIR locus	NSCLC (freq)	Control (freq)	*P* value
3DL1	0.934	0.945	>0.05
2DL1	0.991	1.000	>0.05
2DL3	0.991	0.982	>0.05
2DS4	0.939	0.945	>0.05
2DL2	0.297	0.180	0.064
2DL5	0.441	0.313	0.075
3DS1	0.393	0.336	>0.05
2DS1	0.393	0.332	>0.05
2DS2	0.258	0.180	>0.05
2DS3	0.223	0.143	>0.05
2DS5	0.262	0.286	>0.05
2DL4	1.000	1.000	>0.05
3DL2	1.000	1.000	>0.05
3DL3	1.000	1.000	>0.05
2DP1	0.991	1.000	>0.05
3DP1	1.000	1.000	>0.05

**Notes:** The* P* value is corrected by FDR correction

**Table 5:** * KIR* genotypes frequencies in NSCLC group (n=229) and healthy control group (n=217)

Genotype	NSCLC (freq)	Control (freq)	*P* value
1	0.454	0.512	>0.05
2	0.122	0.088	>0.05
3	0.026	0.023	>0.05
4	0.070	0.037	>0.05
5	0.039	0.005	>0.05
6	0.017	0.009	>0.05
7	0.017	0.009	>0.05
8	0.066	0.074	>0.05
9	0.013	0.014	>0.05
11	0.004	0.000	>0.05
13	0.017	0.014	>0.05
14	0.000	0.014	>0.05
15	0.000	0.028	>0.05
17	0.004	0.005	>0.05
18	0.004	0.000	>0.05
23	0.004	0.037	>0.05
24	0.004	0.005	>0.05
28	0.000	0.005	>0.05
31	0.004	0.000	>0.05
33	0.004	0.000	>0.05
44	0.000	0.014	>0.05
55	0.000	0.005	>0.05
64	0.031	0.000	>0.05
68	0.013	0.023	>0.05
69	0.013	0.009	>0.05
70	0.004	0.005	>0.05
73	0.000	0.005	>0.05
74	0.009	0.000	>0.05
75	0.004	0.014	>0.05
117	0.004	0.000	>0.05
156	0.004	0.000	>0.05
180	0.000	0.014	>0.05
200	0.004	0.000	>0.05
202	0.004	0.014	>0.05
232	0.000	0.005	>0.05
280	0.004	0.000	>0.05
294	0.004	0.000	>0.05
308	0.004	0.000	>0.05
326	0.000	0.005	>0.05
328	0.000	0.005	>0.05
370	0.009	0.000	>0.05
372	0.009	0.000	>0.05
381	0.004	0.000	>0.05
400	0.000	0.009	>0.05

**Notes:** The *P* value is corrected by FDR correction
