# Supplementary material for: The association of HLA/KIR genes with non-small cell lung cancer (adenocarcinoma) in a Han Chinese population
Source: J Cancer. 2019 Aug 20;10(20):4731–8. doi: 10.7150/jca.33566 (PMC6775512; doi:10.7150/jca.33566)
Supplement: Supplementary file 1 — Supplementary tables. [file jcav10p4731s1.pdf]

Supplementary Table 1. KIR Haplotypes frequencies in NSCLC (n=229) and control groups (n=217)

Haplotypes	NSCLC (freq)	Control (freq)	*P* value
AA	0.459	0.525	0.158
Bx	0.541	0.475	

Supplementary Table 2. Distribution of HLA and KIR combination in NSCLC (n=229) and control groups (n=217)

	NSCLC		Control		OR (95% CI)	*P* value
	n	freq.	n	freq.		
KIR3DL2+HLA-A3/A11	112	0.489	118	0.544	0.803 (0.554-1.165)	>0.05
KIR3DL1+HLA-Bw4	109	0.476	117	0.539	0.776 (0.535-1.126)	>0.05
KIR2DL2/3+HLA-C1	219	0.956	206	0.949	1.169 (0.486-2.812)	>0.05
KIR2DL1+HLA-C2	69	0.301	69	0.318	0.925 (0.619-1.382)	>0.05
KIR2DS1+HLA-C2	27	0.118	30	0.138	0.833 (0.477-1.454)	>0.05
KIR2DS2+HLA-C1	55	0.24	38	0.175	1.489 (0.937-2.366)	>0.05

Notes: The *P* value is corrected by FDR correction
